# Endogenous salivary citrate is associated with enhanced rheological properties following oral capsaicin stimulation

**DOI:** 10.1113/EP088166

**Published:** 2019-12-09

**Authors:** Alexander Gardner, Po‐Wah So, Guy Carpenter

**Affiliations:** ^1^ Salivary Research, Centre for Host‐Microbiome Interactions Faculty of Dental, Oral & Craniofacial Sciences King's College London London UK; ^2^ Department of Restorative Dentistry, Dental Hospital and School University of Dundee Dundee UK; ^3^ Department of Neuroimaging, Institute of Psychiatry Psychology and Neuroscience, King's College London Maurice Wohl Clinical Neuroscience Institute London UK

**Keywords:** metabolomics, rheology, saliva

## Abstract

**New Findings:**

- **What is the central question of this study?**
  What are the relationships between physical properties of saliva, protein composition and metabolite composition?
- **What is the main finding and its importance?**
  Salivary citrate, one of the major endogenous metabolites in saliva, increased upon capsaicin stimulation and was associated with improved physical properties measured by extensional rheology. This suggests salivary gland citrate transporters might be a valuable area of future study.

**Abstract:**

Saliva displays viscoelastic properties which enable coating, lubrication and protection of the oral mucosa and hard tissues. Individuals lacking saliva or perceiving oral dryness can manage their symptoms using artificial saliva preparations, but these often fail to mimic the sensation and functionality of natural saliva. It is widely acknowledged that mucins (MUC7 and MUC5B) confer saliva's rheological properties, but artificial saliva containing purified mucins is still often an inadequate substitute. This work aimed to explore salivary components that influence salivary extensional rheology to better understand how natural saliva could be replicated. Saliva was stimulated via control and capsaicin solutions in healthy volunteers. Extensional rheology was analysed using a CaBER‐1 (capillary breakup) extensional rheometer. Protein composition, including mucins, was measured by gel‐electrophoresis band densitometry and metabolites were measured by ^1^H nuclear magnetic resonance spectroscopy. Capsaicin stimulation significantly increased capillary breakup time, extensional viscosity and the abundance of most major salivary proteins. Stimulation also increased salivary citrate and choline concentrations. Significant correlations were found between capillary breakup time and amylase (*r* = 0.67, *P* < 0.05), statherin (*ρ* = 0.66, *P* < 0.05) and citrate (*ρ* = 0.81, *P* < 0.01). The relationship between citrate and salivary rheology was subsequently investigated *in vitro*. These results suggest that citrate and non‐mucin proteins are stronger predictors of salivary rheology than the more often studied mucin glycoproteins. Potential mechanisms are discussed and future work in this area could help formulate more effective saliva substitutes, more closely resembling natural saliva.

## Graphical abstract

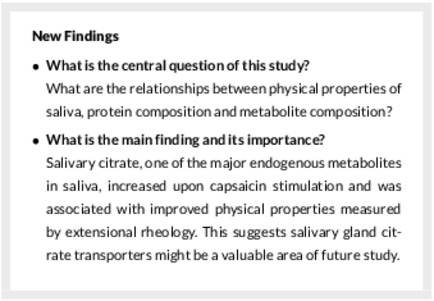

## 1 INTRODUCTION

Saliva serves multiple critical roles in the process of eating. These include facilitating taste perception, mastication, bolus formation and preliminary digestion (Pedersen, Sørensen, Proctor, Carpenter, & Ekström, 2018). Sufferers of hyposalivation (a demonstrable reduction in salivary flow rate) or xerostomia (perceived oral dryness with adequate flow rate, but potentially altered salivary composition) experience a multitude of adverse oral symptoms (Villa, Connell, & Abati, 2015). Consequently, these individuals experience greater risk of nutritional deficiency (Rhodus & Brown, 1990), respiratory infection (Iwabuchi, Fujibayashi, Yamane, Imai, & Nakao, 2012) and potentially all‐cause mortality (Iwasaki et al., 2018).

In order to satisfy functional demands, salivary composition is complex. Whole mouth saliva represents the cumulative output of three paired major glands and several hundred minor glands secreting a fluid composed of water, ions, proteins, metabolites, epithelial cells and bacteria (Humphrey & Williamson, 2001). Of particular importance to salivary function are the physical properties displayed by whole mouth saliva. These include visco‐elastic behaviour allowing lubrication and coating of the oral mucosa. The salivary components primarily responsible for conferring these properties are mucins, high molecular mass glycoproteins that undergo complex structural assembly (Tabak, Levine, Mandel, & Ellison, 1982). There are two salivary mucins, MUC5B and the lower molecular mass MUC7, with each type believed to impact salivary rheology differently. MUC5B has been found to confer viscosity whereas MUC7 appears to confer extensional properties (Inoue et al., 2008).

The exact role of salivary mucins with respect to saliva's physical properties is not fully understood. It is recognised that parotid saliva, which does not contain mucin, lacks the rheological properties of mucin‐rich submandibular and sublingual saliva. However, differences between the physical properties of saliva collected from the latter two glands were not due to differences in mucin content (Van der Reijden, Veerman, & Nieuw Amerongen, 1993). Similarly, mucin content alone fails to explain rheological differences measured in chewing‐stimulated submandibular and sublingual saliva (Vijay, Inui, Dodds, Proctor, & Carpenter, 2015). What is apparent is that replicating the physical properties of natural saliva *ex vivo* is an ongoing challenge (Hanchanale, Adkinson, Daniel, Fleming, & Oxberry, 2015). In one trial of xerostomia patients, it was found that four times as many patients preferred using chewing gum to stimulate their own saliva than using artificial saliva (Bots et al., 2005). Purified mucin solutions have been shown to lack the physical properties of saliva (Rossetti, Yakubov, Stokes, Williamson, & Fuller, 2008). Commercially available saliva substitutes with purified mucin formulations are available, but studies indicate they are no better received than mucin‐free placebos, and natural saliva stimulation by chewing gum is still preferable (Davies, 2000; Sweeney, Bagg, Baxter, & Aitchison, 1997).

Furthermore, the physical properties of natural saliva are short‐lived outside the mouth (Houghton et al., 2017), and physical modification of saliva, such as centrifugation, also causes a loss of rheological properties (Haward, Odell, Berry, & Hall, 2011). While there is no consensus on which rheological measurements offer the greatest biological or clinical relevance, it is widely agreed that for rheological purposes samples must be analysed as rapidly as possible after collection, without modification. Literature on salivary rheology includes bulk rheology (Davies, Wantling, & Stokes, 2009; Schwarz, 1987; Stokes & Davies, 2007) and extensional rheology. Typically, salivary extensional rheology measures spinnbarkeit, the filament forming ability of the fluid (Chaudhury, Shirlaw, Pramanik, Carpenter, & Proctor, 2015; Gohara et al., 2004; Vijay et al., 2015). Alternative measures of salivary extensional rheology include capillary breakup (thinning rate of a stretched capillary of fluid) (Turcanu, Tascon, Balan, & Gallegos, 2015; Zussman, Yarin, & Nagler, 2007), as well as extensional flow oscillatory rheology (Haward et al., 2011). The former has also been applied to the study of saliva–food mixtures, which could have important implications for mastication and deglutition (Choi, Mitchell, Gaddipati, Hill, & Wolf, 2014). Extensional rheology offers the advantage of lower sample volume and more rapid data acquisition.

Stimulation of saliva production can modify its rheological properties thereby providing an alternative approach to artificial saliva for managing dry mouth symptoms. Stokes and Davies (2007) found that citric acid‐stimulated saliva was more viscous than mastication stimulated saliva when matched for flow rate. This was attributed to greater activation of minor salivary glands by citric acid gustatory reflexes resulting in a more proteinaceous saliva (Stokes & Davies, 2007). An important consideration is that substances such as acids are known to have a direct chemical effect on saliva independent from, or in addition to, their gustatory properties. Investigation of such processes is beneficial in interpreting the interactions of saliva and citric acid. Houghton *et al*. reported increased spinnbarkeit following salivary stimulation by the transient receptor potential (TRP) agonist nonivamide. This was also hypothesised to be due to a modification of the protein content of the stimulated saliva. The authors identified nonivamide as potentially useful in management of dry mouth based on the observed effects on salivary rheology (Houghton et al., 2017). Nonivamide is a capsaicin analogue. Capsaicin is a molecule found in spicy foods that activates TRPV1 channels in the mouth, causing a sensation of heat. Capsaicin has also been identified as a modulator of salivary composition, and has therapeutic potential in managing oral disease (Kono, Kubota, Taira, Katsuyama, & Sugimoto, 2018). While stimulation by 3% citric acid has been shown to improve dry‐mouth symptoms (Femiano et al., 2011), the use of citric acid in dentate individuals is contraindicated due to its damaging erosive effects on remaining teeth (Shaw et al., 2000). Vijay *et al*. found olfaction and monosodium glutamate (umami) taste also increase salivary spinnbarkeit and interfacial rheology with accompanying protein changes. Furthermore, they reported spinnbarkeit was associated with salivary bicarbonate and calcium concentration. Thus, protein composition is not the sole compositional factor mediating the physical properties of saliva (Vijay et al., 2015).

This study aimed to explore changes in salivary protein, metabolite composition and extensional rheology following stimulation using capsaicin (an analogue of nonivamide) in order to better understand the compositional factors that drive the physical properties of saliva. Relationships between biochemical and physical changes were assessed.

## 2 METHODS

### 2.1 Ethical approval

All research followed ethical approval from King's College London ethics committee (HR‐15/15‐2508). Research was conducted in accordance with the *Declaration of Helsinki*, except for registration in a publicly accessible database, and written informed consent of participants was obtained.

### 2.2 Tastant preparation

Bottled water (Buxton), food‐grade ethanol (Sigma‐Aldrich, Gillingham, UK) and pharmaceutical grade capsaicin (USP, Rockville, MD, USA) were purchased. As capsaicin is insoluble in water, it was pre‐dissolved in ethanol then diluted with water to solutions of final concentration of 1 parts per million (ppm) capsaicin and 0.095% ethanol by volume. A control solution of 0.095% ethanol was also prepared. All samples were prepared fresh prior to the experiment from the same stock solution.

### 2.3 Sample collection

Participants were 10 healthy adults (five male) with no active oral pathology. Participants abstained from oral exposure to exogenous substances (eating, chewing gum, smoking or oral hygiene) for 1 h prior to sample collection.

Participants passively held 10 ml of a pre‐weighed control solution in the mouth for 30 s, then expectorated the solution into the same container. The solution was re‐weighed to calculate in‐mouth flow rate.

Saliva was then collected over a period of 2 min by spitting into pre‐weighed, sterilised universal tubes. Flow rate was calculated in grams per minute by re‐weighing the tube after saliva collection.

There was a 10 min rest period while rheological measurements were conducted as described below. This saliva collection process was repeated for the capsaicin solution.

### 2.4 Salivary analyses

#### 2.4.1 Extensional rheology

Samples were immediately analysed using a HAAKE CaBER‐1 extensional rheometer (Thermo Fisher Scientific, Waltham, MA, USA). The sample was loaded between 6 mm diameter plates set at an initial gap of 2 mm, requiring 56.5 µl of sample. To avoid introducing bubbles when loading, 60 µl of sample was pipetted to ensure a slight excess remained in the pipette tip. Capillary formation was set to a sample end height of 10.8 mm with a stretch time of 50 ms. Three measurements were made per sample and averaged. Total capillary breakup time and apparent extensional viscosity over a range of Hencky strains (9, 9.5, 10 and 10.5) were analysed. Sample surface tension was set to 52 mN m^−1^ based on literature values (Kazakov, Udod, Zinkovych, Fainerman, & Miller, 2009; Vijay et al., 2015). Sample density was set to 1 g cm^−3^.

As the CaBER requires a fresh sample for each reading, the plates were cleaned between readings with distilled water. Between participants, the rheometer plates were cleaned with ethanol followed by distilled water and then air dried.

Following rheological analysis, residual saliva was centrifuged at 15,000 *g* for 10 min at 4°C and aliquots of supernatant were frozen at −80°C for protein and metabolic compositional analysis.

#### 2.4.2 Protein semi‐quantification

**Figure 1:**
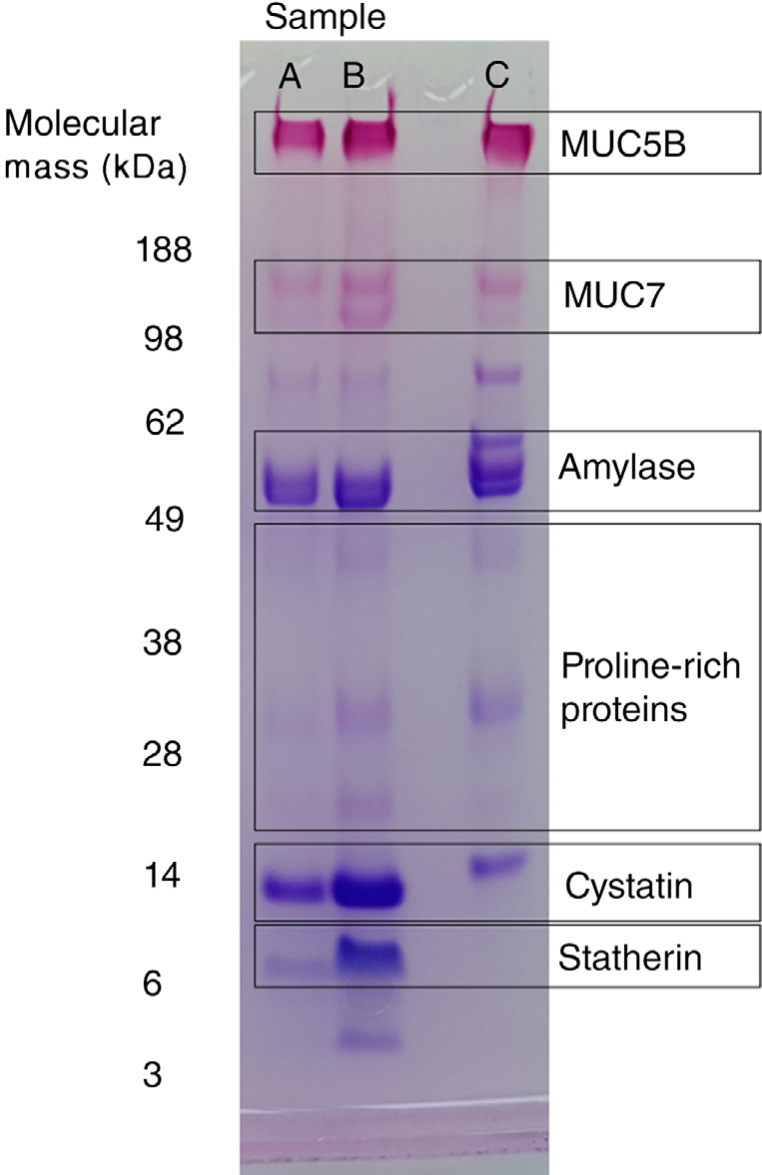
Representative Coomassie and periodic acid–Schiff stained polyacrylamide gel. Lanes A, B and C are loaded with equal volumes of control stimulated saliva (A), capsaicin stimulated saliva (B) and the standard reference saliva sample (C). The protein bands measured are labelled. Note for proline‐rich proteins the prominent central band (between 38 and 28 kDa) was measured

The relative abundance of major salivary proteins in each sample was semi‐quantified by gel densitometry of stained SDS‐PAGE gels, as described by Chaudhury et al. (2015). Briefly, one part lithium dodecyl sulphate buffer (Thermo Fisher Scientific, Carlsbad, CA, USA) was added to three parts sample supernatant. Dithiothreitol (0.5 m) was added to a concentration of 10% and the solution was boiled for 3 min. Sample mixture (10 µl) was loaded into 4–12% Bis‐Tris polyacrylamide gels (Thermo Fisher Scientific) and electrophoresed at 200 V, 250 mA. A standardised reference saliva sample was run on every gel. Gels were then fixed and stained in Coomassie Brilliant Blue R250 (Sigma‐Aldrich, Gillingham, UK) with 25% methanol for 30 min and de‐stained in 10% glacial acetic acid and deionised water. Gels were imaged with a ChemiDoc MP imaging system (Bio‐Rad, Hemel Hempstead, UK). Gels were then oxidised in 2% periodic acid solution for 15 min, washed three times (10 mins) in deionised water and stained with Schiff's reagent (VWR, Lutterworth, UK) for 30 min to visualise glycoproteins (MUC7 and MUC5B). Gels were destained with deionised water and re‐imaged. Band intensity of major proteins was semi‐quantified relative to the standard sample cystatin band for non‐mucins and MUC5B band for mucins with ImageLab 4.0 software (Bio‐Rad). A representative gel showing the proteins measured is shown in Figure 1.

#### 2.4.3 Metabolite profiling by ^1^H‐NMR spectroscopy

Sample preparation, spectral acquisition and spectral processing were in accordance with a previously described protocol (Gardner, Parkes, Carpenter, & So, 2018). Briefly, samples were prepared in a 4:1 ratio with 0.5 mm trimethylsilyl‐[2,2,3,3,‐^2^H_4_]‐propionate (TSP) internal standard and 10% deuterium oxide (Sigma‐Aldrich). Solutions were analysed in 3 mm o.d. NMR tubes (Sigma‐Aldrich).

Samples underwent ^1^H nuclear magnetic resonance (NMR) spectroscopy on a NMR spectrometer (Bruker, Karlsruhe, Germany) at a proton frequency of 600.2 MHz at 25°C. A Carr–Purcell–Meiboom–Gill (CPMG) spin‐echo pulse sequence with water presaturation was used to filter out resonances from residual macromolecules. Samples were analysed in automation after a single freeze–thaw cycle and maintained at 4°C when queued for analysis. A full list of metabolites quantified is present in Table 1.

**Table 1:** List of salivary metabolite concentrations following control and capsaicin stimulation

	Metabolite concentration post‐control (mm)		Metabolite concentration post‐capsaicin (mm)			
Metabolite	Mean	SD	Mean	SD	Mean fold change	Significance (*P*‐value)
Citrate	0.020	0.015	0.048	0.050	2.46	***0.004***
Phenylalanine	0.022	0.011	0.029	0.017	1.32	*0.36*
Histidine	0.009	0.005	0.011	0.008	1.26	0.14
Butyrate	0.128	0.053	0.157	0.080	1.23	0.11
Dimethylamine	0.011	0.006	0.013	0.008	1.22	0.12
Alanine	0.051	0.031	0.061	0.048	1.21	*0.13*
Tyrosine	0.018	0.012	0.021	0.012	1.18	0.06
Acetoin	0.025	0.015	0.029	0.018	1.15	0.16
Choline	0.016	0.011	0.017	0.012	1.10	**0.03**
Pyruvate	0.054	0.030	0.059	0.034	1.10	0.18
Trimethylamine	0.003	0.003	0.004	0.004	1.09	0.51
Succinate	0.089	0.081	0.096	0.080	1.08	0.49
Methylamine	0.003	0.002	0.003	0.003	1.07	0.47
Acetate	2.27	1.52	2.20	1.74	1.01	0.77
Glycine	0.075	0.081	0.075	0.083	1.00	0.39
Methanol	0.029	0.013	0.029	0.013	0.98	0.71
Taurine	0.051	0.035	0.048	0.037	0.96	0.67
Lactate	0.097	0.051	0.093	0.052	0.95	0.65
Formate	0.042	0.062	0.037	0.053	0.88	*0.45*
Propionate	0.341	0.260	0.291	0.267	0.85	0.13
Urea	0.152	0.109	0.119	0.066	0.78	0.13

Metabolites are ranked by fold change, and significant changes are denoted by bold text. Values in italics are from Wilcoxon signed‐rank tests, otherwise a paired *t* test was conducted. *n* = 10.

#### 2.4.4 Validation of citrate quantification by ^1^H‐NMR using enzymatic assay

Saliva samples were collected from an additional seven participants who had not previously completed the study. Samples were collected post‐control and post‐capsaicin stimulation as described. Samples were analysed by ^1^H‐NMR as described. The citrate concentration of each sample was also measured using a fluorometric citrate assay kit (Sigma‐Aldrich), and results between the two methods were compared.

#### 2.4.5 *In vitro* rheological analysis of effect of exogenous citric acid on saliva

We observed that saliva expectorated after administering an oral solution contains approximately 10% of the original solution based on the solvent content of capsaicin solutions pre‐ and post‐expectoration (16.2 to 1.16 mm). Stokes and Davies (2007) administered up to 1% citric acid solutions to stimulate saliva, and thus their collected saliva was estimated to contain approximately 0.1% citric acid.

Unstimulated saliva (∼ 1.5 ml) was collected from volunteers as described and capillary breakup time was measured. Saliva was then mixed with 1% citric acid in deionised water (450 µl: 50 µl), yielding a final citric acid concentration of 0.1%. A separate 450 µl of saliva was mixed in the same ratio with pure deionised water as a control. Capillary breakup time was re‐assessed for both samples.

### 2.5 Statistical analyses

Statistical analysis was conducted in SPSS Statistics Version 24 (IBM Corp., Armonk, NY, USA). All data were inspected for normality by Q‐Q plots and the Shapiro–Wilk test. Differences in capsaicin/control stimulated flow rate, rheological measurements and compositional changes were assessed by the Wilcoxon signed‐rank test or Student's paired *t* test where appropriate. Relationships between rheological measurements and salivary components for all samples were assessed based on normality by appropriate correlation tests (Spearman's rank or Pearson's) and regression analyses (linear or non‐linear).

Capillary breakup times of samples in the *in vitro* experiment were compared by Dunn's multiple comparison test following Friedman's test. For completeness, salivary data post‐control and post‐capsaicin are presented in Tables 2 and 3, respectively.

**Table 2:** Summary of all measured data in saliva samples following control stimulation

	Participant number									
Parameter	1	2	3	4	5	6	7	8	9	10
In mouth flow rate (g min^−1^)	−0.70	0.28	1.19	‐0.21	1.12	0.64	1.44	0.59	−0.89	−0.68
Flow rate post stimulus (g min^−1^)	0.71	0.70	1.03	1.00	1.04	0.75	1.11	1.52	1.22	1.74
Total protein (mg ml^−1^)	1.55	0.83	0.81	1.42	0.85	0.96	0.74	0.87	1.26	0.89
Amylase (relative intensity)	2.56	1.54	1.67	1.84	1.08	1.89	1.04	1.31	1.26	1.03
Proline‐rich protein (relative intensity)	0.10	0.19	0.33	0.67	0.08	0.10	0.11	0.28	0.50	0.27
Cystatin (relative intensity)	0.54	1.08	0.30	0.72	0.22	0.34	0.42	0.68	0.92	0.71
Statherin (relative intensity)	0.31	0.14	0.11	0.39	0.01	0.03	0.02	0.12	0.26	0.13
MUC7 (relative intensity)	0.03	0.06	0.09	0.21	0.02	0.08	0.02	0.00	0.11	0.12
MUC5B (relative intensity)	0.48	0.78	0.37	0.25	0.18	0.46	0.35	0.25	0.48	0.55
Formate (mm)	0.070	0.012	0.017	0.013	0.033	0.020	0.010	0.007	0.027	0.212
Histidine (mm)	0.013	0.009	0.006	0.017	0.000	0.008	0.004	0.005	0.014	0.011
Phenylalanine (mm)	0.025	0.038	0.018	0.027	0.000	0.021	0.011	0.016	0.029	0.036
Tyrosine (mm)	0.023	0.028	0.015	0.011	0.000	0.018	0.009	0.011	0.022	0.043
Urea (mm)	0.383	0.310	0.119	0.133	0.080	0.035	0.066	0.134	0.145	0.115
Glycine (mm)	0.084	0.049	0.032	0.046	0.021	0.049	0.013	0.028	0.160	0.271
Taurine (mm)	0.070	0.095	0.024	0.043	0.012	0.023	0.022	0.027	0.102	0.088
Methanol (mm)	0.041	0.025	0.035	0.012	0.013	0.016	0.033	0.029	0.044	0.047
Choline (mm)	0.027	0.026	0.011	0.022	0.002	0.007	0.005	0.007	0.036	0.012
Trimethylamine (mm)	0.004	0.005	0.004	0.000	0.000	0.004	0.003	0.000	0.009	0.007
Dimethylamine (mm)	0.017	0.017	0.010	0.018	0.003	0.007	0.005	0.004	0.013	0.014
Methylamine (mm)	0.005	0.007	0.003	0.000	0.000	0.003	0.003	0.000	0.005	0.000
Citrate (mm)	0.043	0.021	0.023	0.043	0.008	0.012	0.010	0.006	0.000	0.030
Succinate (mm)	0.124	0.051	0.064	0.117	0.048	0.043	0.039	0.016	0.090	0.298
Pyruvate (mm)	0.092	0.068	0.034	0.077	0.018	0.031	0.032	0.022	0.069	0.097
Acetate (mm)	2.209	2.019	2.003	1.344	1.104	2.130	1.093	0.961	4.086	5.740
Lactate (mm)	0.138	0.178	0.075	0.102	0.045	0.054	0.090	0.021	0.107	0.161
Propionate (mm)	0.364	0.216	0.302	0.060	0.142	0.560	0.128	0.148	0.662	0.829
Butyrate (mm)	0.181	0.109	0.125	0.102	0.060	0.153	0.064	0.084	0.196	0.203
Alanine (mm)	0.070	0.033	0.035	0.070	0.022	0.031	0.023	0.023	0.096	0.103
Acetoin (mm)	0.036	0.033	0.014	0.022	0.013	0.015	0.017	0.010	0.034	0.056
Ethanol (mm)	11.617	4.766	4.629	1.412	2.459	2.668	1.549	1.877	3.751	5.714
Capillary breakup time (s)	2.13	2.26	4.17	1.30	0.30	0.42	0.69	0.00	0.09	1.00
Apparent extensional viscosity at strain 9 (mPa s)	65.0	21.0	5.25	8.60	7.0	13.0	16.50	0.0	1.75	13.80
Apparent extensional viscosity at strain 9.5 (mPa s)	172.0	50.0	7.17	18.0	8.0	17.20	20.50	0.0	2.50	16.50
Apparent extensional viscosity at strain 10 (mPa s)	290.0	90.0	9.80	34.0	9.70	18.60	23.60	0.0	3.10	27.50
Apparent extensional viscosity at strain 10.5 (mPa s)	418.0	142.0	13.39	75.60	11.80	25.00	28.50	0.0	7.00	53.50

**Table 3:** Summary of all measured data in saliva samples following capsaicin stimulation

	Participant number									
Parameter	1	2	3	4	5	6	7	8	9	10
In mouth flow rate (g min^−1^)	0.73	1.40	2.68	1.35	1.64	0.33	1.86	1.05	1.80	1.12
Flow rate post stimulus (g min^−1^)	0.99	1.29	2.15	0.73	0.84	1.01	1.53	1.43	1.35	1.70
Total protein (mg ml^−1^)	1.30	1.40	1.11	2.01	0.76	1.29	0.99	1.35	1.27	1.09
Amylase (relative intensity)	2.94	2.11	2.02	2.01	1.37	1.97	1.40	1.44	1.14	1.25
Proline‐rich protein (relative intensity)	0.15	0.34	0.43	0.83	0.10	0.11	0.15	0.45	0.49	0.40
Cystatin (relative intensity)	1.23	2.75	0.79	1.80	0.47	1.04	1.04	1.20	0.96	1.24
Statherin (relative intensity)	0.80	2.01	0.30	0.85	0.01	0.13	0.08	0.64	0.54	0.54
MUC7 (relative intensity)	0.05	0.14	0.12	0.20	0.04	0.08	0.08	0.00	0.11	0.20
MUC5B (relative intensity)	0.60	1.06	0.46	0.18	0.17	0.46	0.43	0.28	0.50	0.89
Formate (mm)	0.045	0.017	0.014	0.015	0.022	0.018	0.010	0.006	0.039	0.183
Histidine (mm)	0.013	0.013	0.006	0.020	0.000	0.007	0.003	0.004	0.027	0.014
Phenylalanine (mm)	0.034	0.047	0.017	0.047	0.000	0.027	0.015	0.014	0.046	0.042
Tyrosine (mm)	0.027	0.031	0.010	0.019	0.000	0.020	0.011	0.021	0.031	0.043
Urea (mm)	0.240	0.204	0.075	0.075	0.081	0.092	0.050	0.00	0.163	0.094
Glycine (mm)	0.084	0.044	0.023	0.045	0.015	0.050	0.012	0.031	0.195	0.255
Taurine (mm)	0.061	0.069	0.017	0.032	0.015	0.026	0.017	0.039	0.133	0.077
Methanol (mm)	0.039	0.023	0.028	0.013	0.009	0.017	0.034	0.036	0.049	0.038
Choline (mm)	0.032	0.029	0.014	0.024	0.002	0.010	0.006	0.007	0.035	0.012
Trimethylamine (mm)	0.004	0.003	0.003	0.000	0.000	0.005	0.003	0.000	0.012	0.007
Dimethylamine (mm)	0.017	0.018	0.008	0.025	0.003	0.009	0.005	0.004	0.026	0.016
Methylamine (mm)	0.006	0.005	0.003	0.000	0.000	0.004	0.002	0.000	0.006	0.000
Citrate (mm)	0.075	0.065	0.026	0.176	0.009	0.024	0.026	0.031	0.000	0.049
Succinate (mm)	0.125	0.068	0.067	0.128	0.037	0.049	0.031	0.017	0.159	0.280
Pyruvate (mm)	0.094	0.088	0.035	0.077	0.018	0.037	0.031	0.021	0.102	0.092
Acetate (mm)	2.096	1.633	1.136	1.387	0.879	2.108	1.013	1.012	5.794	4.945
Lactate (mm)	0.134	0.162	0.049	0.108	0.030	0.071	0.052	0.034	0.166	0.123
Propionate (mm)	0.288	0.157	0.126	0.032	0.106	0.443	0.099	0.169	0.818	0.671
Butyrate (mm)	0.192	0.179	0.091	0.228	0.043	0.154	0.068	0.108	0.298	0.213
Alanine (mm)	0.070	0.040	0.040	0.088	0.015	0.039	0.021	0.029	0.170	0.100
Acetoin (mm)	0.037	0.038	0.012	0.030	0.009	0.021	0.017	0.015	0.059	0.055
Ethanol (mm)	3.754	2.425	1.415	0.929	1.942	2.419	1.295	1.485	2.518	4.665
Capillary breakup time (s)	4.87	4.72	4.99	5.95	0.02	1.03	1.86	2.05	0.54	3.16
Apparent extensional viscosity at strain 9 (mPa s)	134.0	150.0	8.1	35.0	2.94	16.00	5.05	5.15	3.50	52.0
Apparent extensional viscosity at strain 9.5 (mPa s)	359.0	212.0	42.0	214.0	4.11	21.45	6.0	5.40	4.90	265.0
Apparent extensional viscosity at strain 10 (mPa s)	635.0	410.0	208.0	408.0	5.27	21.75	80.0	5.70	9.0	577.0
Apparent extensional viscosity at strain 10.5 (mPa s)	1250.0	683.0	554.0	585.5	6.43	25.30	281.0	5.98	19.0	870.0

## 3 RESULTS

### 3.1 Capsaicin stimulation increases salivary capillary breakup time and apparent extensional viscosity

**Figure 2:**
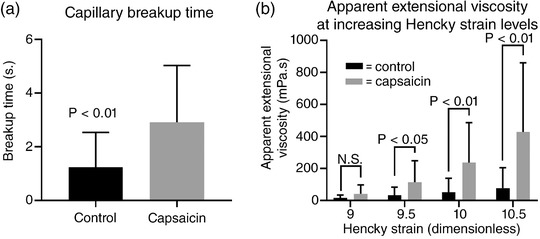
Summary of the extensional rheological properties of capsaicin‐stimulated saliva. (a) Capsaicin stimulation significantly increased capillary breakup time by approximately three‐fold. (b) There is a trend for increased relative apparent extensional viscosity at increasing Hencky strain upon capsaicin stimulation relative to control. Stimulated viscosity increases were all significant except viscosity at Hencky strain 9. Statistical tests are paired *t* test (capillary breakup) and Wilcoxon signed‐rank test (viscosity), *n* = 10. N.S., not significant. Data are shown as means ± standard deviation (SD)

Capillary breakup and apparent extensional viscosity at Hencky strains 9.5, 10 and 10.5 were significantly higher for capsaicin stimulated saliva (Figure 2). At increasingly higher strains the differences in viscosities between the control and capsaicin‐stimulated samples were greater in magnitude. This relationship suggests saliva is strain‐hardening, consistent with other literature findings (Wagner & McKinley, 2017). The difference in magnitude post‐capsaicin may reflect the greater polymer load (i.e. salivary protein) present in the sample.

### 3.2 Capsaicin stimulation increases salivary flow rate and abundance of major salivary proteins

**Figure 3:**
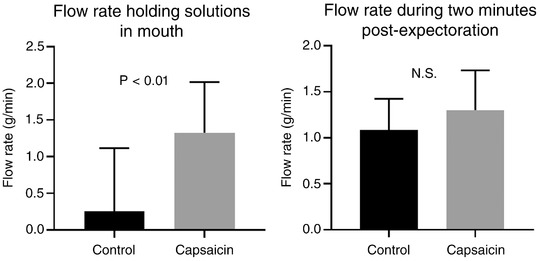
Mean salivary flow rate was significantly higher whilst capsaicin was held in the mouth. Flow rate differences in the 2 min post‐stimulation were not statistically significant (paired *t* test, *n* = 10). Data are shown as means ± SD. Note, the error bar spanning zero for the control solution results from a ‘negative’ flow rate in some participants, indicative of a net fluid loss following the rinse, due to coating of liquid onto oral tissues

Salivary flow rate was significantly increased by capsaicin stimulation whilst held in the mouth, but increases were not sustained into the subsequent 2 min saliva collection period, Figure 3.

**Figure 4:**
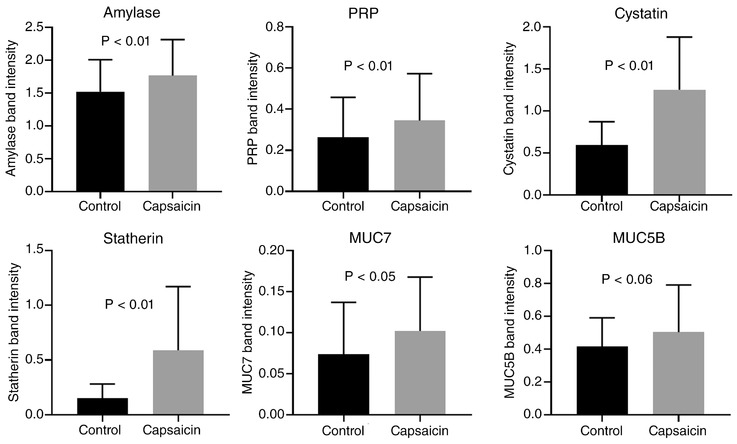
Summary of changes in salivary protein band intensity following control and capsaicin stimulation. *P*‐values by paired *t* tests, *n* = 10. Data are shown as means ± SD

Major salivary proteins measured by band densitometry were MUC5B, MUC7, amylase, PRP, cystatin and statherin. Band assignments were made relative to molecular mass as described previously (Carpenter, 2013; Gardner & Carpenter, 2019). The relative band intensities of salivary proteins were increased following capsaicin stimulation. The differences were significant in all cases except for MUC5B where the *P*‐value approached the 0.05 threshold of significance (Figure 4). The largest fold change in intensity was for the statherin band, which underwent a threefold increase on average, followed by the cystatin band, which approximately doubled in intensity.

### 3.3 Capsaicin stimulation increases salivary citrate concentration

**Figure 5:**
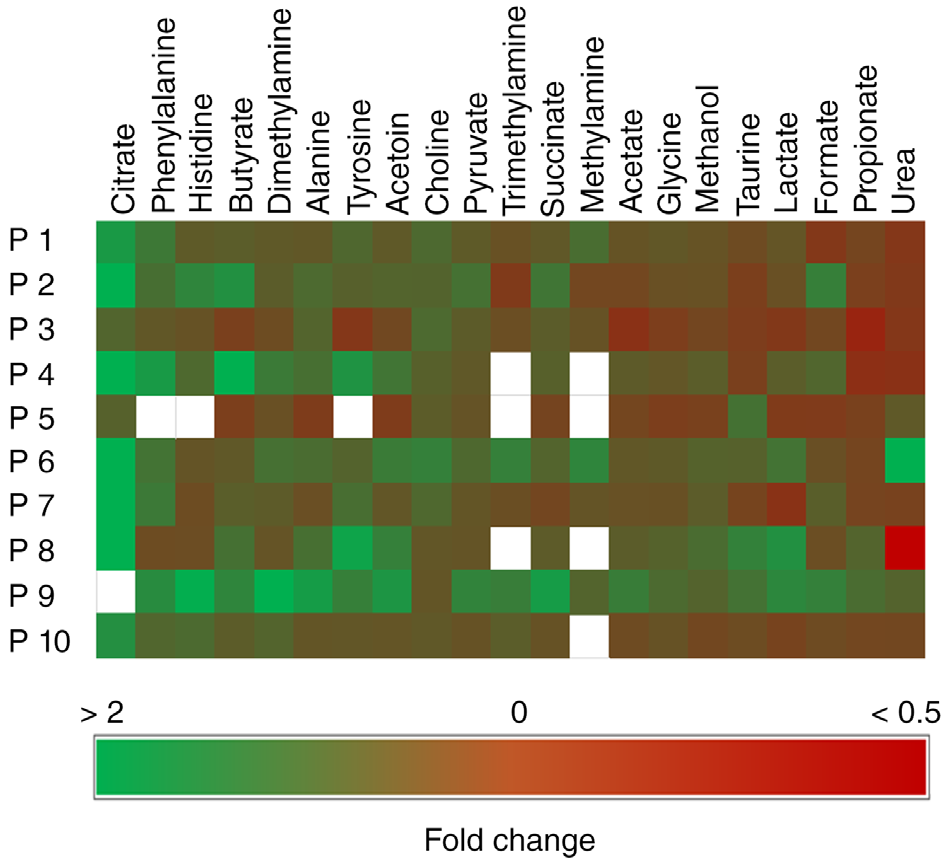
Heatmap of the salivary metabolite concentration fold changes following control and capsaicin stimulation for the 10 participants. Data are ranked by largest to smallest mean metabolite fold change. Blank boxes represent instances where a metabolite was absent/not quantifiable from the control saliva meaning relative change could not be calculated due to division by zero

Concentrations of the majority of salivary metabolites quantified were not significantly different following capsaicin stimulation (Table 1). Fold changes at an individual level are summarised in the heatmap shown in Figure 5.

**Figure 6:**
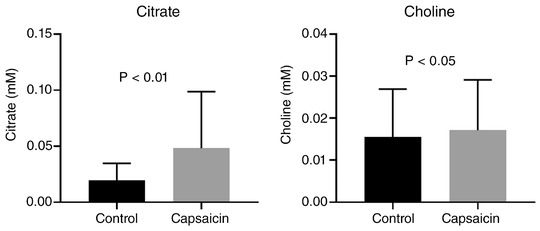
Summary of salivary metabolites where concentration differed significantly following capsaicin stimulation. Citrate data are analysed by Wilcoxon signed‐rank test and choline data by paired *t* test, both (*n* = 10). Data are means ± SEM

Statistically significant changes were observed for citrate and to a lesser extent choline, both of which increased following stimulation (Figure 6).

**Figure 7:**
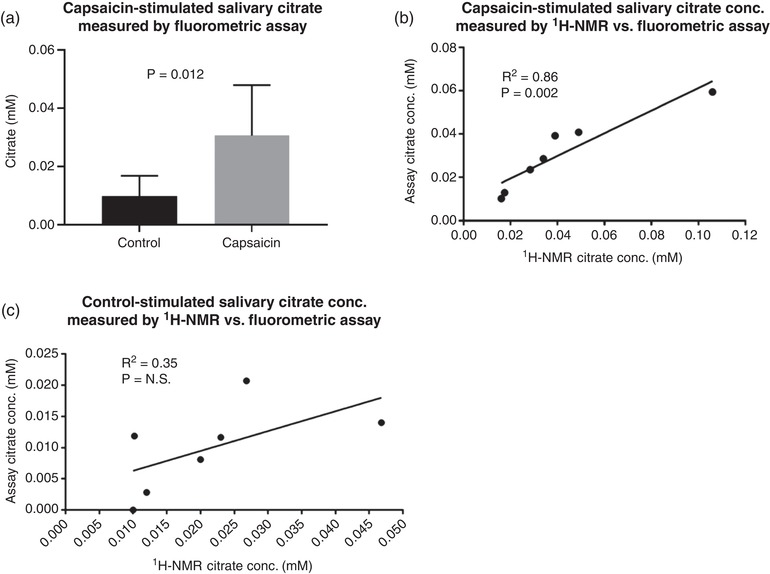
Validation of ^1^H‐NMR quantification of salivary citrate. (a) A statistical increase in salivary citrate was confirmed when measuring citrate via fluorometric assay. (b) The relationship between capsaicin‐stimulated salivary citrate measured by ^1^H‐NMR and by fluorometric assay. The relationship appears linear, although slightly offset by the highest concentration sample, which weakened the strength of this relationship. (c) Lack of relationship between control‐stimulated salivary citrate measured by ^1^H‐NMR and by fluorometric assay, possibly due to the citrate concentrations being close to the assay limit of detection. Data were analysed by paired *t* test and Pearson's correlation (*n* = 7). Data in (a) are means ± SD, N.S., not significant

Significant capsaicin‐stimulated increase in salivary citrate were confirmed when measured by fluorometric assay. Citrate concentration measured by assay and ^1^H‐NMR correlated strongly for capsaicin‐stimulated samples but not for control samples, likely due to the citrate concentrations in the control samples being close to the limit of detection of the assay. Data are shown in Figure 7.

### 3.4 Salivary citrate, amylase and statherin are associated with rheological measurements

**Figure 8:**
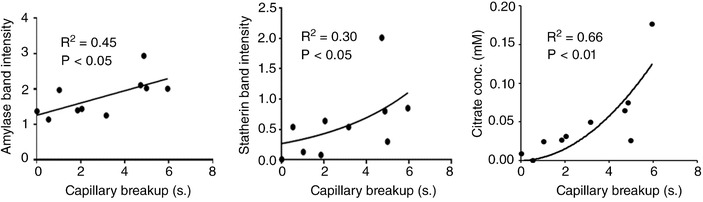
Summary of significant relationships between capillary breakup and salivary components (amylase, statherin and citrate). Linear regression analysis was performed for amylase whereas statherin and citrate were analysed by nonlinear regression

In capsaicin‐stimulated samples significant correlations between capillary breakup time and protein composition were observed for amylase (*r* = 0.67, *P* = 0.035; Figure 8) and statherin (*ρ* = 0.66, *P* = 0.037; Figure 8). The only metabolite found to correlate with rheological properties was citrate which displayed a relatively strong association with capillary breakup time (*ρ* = 0.81, *P* = 0.005; Figure 8).

In control‐stimulated saliva, no significant relationships were observed between rheological parameters and the measured salivary components.

### 3.5 Endogenous citric acid alters salivary rheology independent from gustatory reflexes

**Figure 9:**
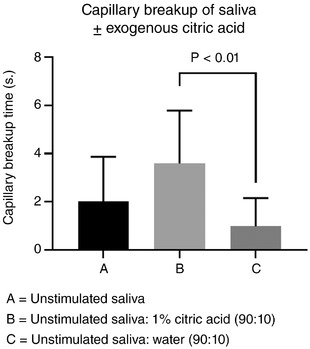
Comparison of unstimulated saliva mixed outside the mouth 90:10 with 1% citric acid or water. Data are means ± SD; *P*‐values are from Dunn's multiple comparison test following Friedman's test (*n* = 6)

Comparison of the *in vitro* effects of citric acid on saliva are shown in Figure 9. Saliva mixed 9:1 with 1% citric acid showed a significantly increased capillary breakup time compared to saliva mixed with water at the same ratio. This is indicative of an effect of citric acid on salivary rheology independent of reflex gustatory salivation. The largest capillary breakup time observed was 6.55 s despite the 0.1% (5.2 mm) citric acid concentration. This confirms the non‐linear relationship between citrate concentration and capillary breakup time suggested in Figure 8, as capillary breakup time reached a limit that was not increased by further increasing citrate concentration.

## 4 DISCUSSION

This study found that capsaicin stimulation brought about significant increases in both the extensional rheology of saliva and the abundance of major salivary proteins. The amount of amylase and statherin showed significant relationships with extensional rheology measured by capillary breakup time. These findings support the hypothesis that following TRPV1 channel activation by substances such as capsaicin, changes in the protein composition in saliva may be responsible for changes in salivary extensional rheology (Houghton et al., 2017).

While MUC7 was significantly increased following capsaicin stimulation, no relationship between mucins and extensional rheology was found. Other studies have implicated MUC7 in particular in conferring spinnbarkeit to saliva (Vijay et al., 2015; Inoue et al., 2008). The lack of relationship observed between mucin concentration and capillary breakup time may reflect differences in rheological measurement between spinnbarkeit and capillary breakup. The finding that amylase and statherin related to salivary capillary breakup time may well support the concept of non‐mucins having a role in the assembly of mucin complexes (Tabak, 1995). While amylase displayed relatively small but consistent increases following capsaicin stimulation, statherin showed on average a threefold increase. Statherin is known to undergo breakdown by bacterial proteases in the oral cavity (Helmerhorst, Traboulsi, Salih, & Oppenheim, 2010), and therefore could be considered a marker of the ‘freshness’ of the protein content of saliva. This may be important given the rheological changes in stimulated saliva appear to reduce rapidly, within minutes of stimulation (Houghton et al., 2017). This would imply that in the same way salivary rheology breaks down *in vitro*, saliva secreted into the mouth will slowly lose the rheological properties initially present at the point of secretion. This would partially explain why mucins in artificial saliva preparations are generally not an adequate replacement for natural saliva.

To our knowledge this is the first study to profile salivary metabolites with respect to physical properties of saliva. While the majority of metabolites did not change significantly, citrate showed a large significant increase. Choline also significantly increased, although to a lesser extent with a small but consistent increase across participants. The choline peak also encompasses phosphorylcholine, which we have previously shown was associated with the cellular content of saliva as it is an important constituent of cell membranes (Gardner et al., 2018). The observed increase in choline may therefore be related to a small increase in the number of epithelial cells shed into saliva possibly due to oral movement or increased flow rate during capsaicin tasting. The increase in salivary citrate was an interesting finding. We have previously found that citrate is one of a few host‐derived metabolites present in saliva (Gardner, Parkes, So, & Carpenter, 2019). Additionally, unlike other host derived metabolites such as urea and lactate, the citrate concentration of parotid saliva does not reduce upon salivary stimulation, suggesting the molecule is generated at the same rate as fluid production. Citrate is known to be an important metabolite with a range of biological functions. While specific transporters of citrate of the family SLC13 are present in epithelial cells composing many tissues and organs, their presence in salivary glands in unclear. Pathological conditions of organs such as the prostate gland, which is specialised in concentrating citrate, are associated with deficiencies in citrate transport (Mycielska et al., 2009). Drug development targeting these transporters is ongoing (Colas, Ung, & Schlessinger, 2016). Defective citrate transport could be an avenue to explore in the aetiology of dry‐mouth, particularly xerostomia where flow rate is normal but salivary function may be inadequate.

Citrate was also found to have the strongest relationship with extensional rheology out of all measured salivary components. Possible explanations for this observation may relate to the ionic environment of salivary proteins. Salivary proteins are negatively charged and prior to secretion are packaged with abundant calcium ions (divalent cations) to restore a net charge balance (Proctor, 2016). The initial unfolding of secreted gastric mucins has been related to localised decreases in ions including calcium (Meldrum et al., 2018). In mucins of the respiratory tract, this process has been shown to be influenced by bicarbonate, with bicarbonate chelating calcium ions and facilitating mucin functionality (Chen, Yang, Quinton, & Chin, 2010). Salivary bicarbonate has been demonstrated to inversely relate to spinnbarkeit (Vijay et al., 2015). Salivary citrate is also known to be one of the main calcium chelators in saliva (Silwood, Grootveld, & Lynch, 2002), and therefore could be equally important in aiding mucin assembly.

Exogenous citric acid has also been shown to increase the bulk viscosity of saliva, but this was attributed to the stimulant properties of citric acid which would not likely occur at the low concentrations of endogenous citrate in saliva (Stokes & Davies, 2007). Furthermore, the changes in rheology following citric acid stimulation may be due to post‐secretion chemical interactions between saliva and citric acid, as demonstrated in Figure 9. The association between citrate and capillary breakup time shown in Figure 8 implies a threshold citrate concentration above which capillary breakup time no longer increases. This non‐linear relationship is supported by the data in Figure 9, where exogenous citrate 100‐fold greater than salivary levels confers only a slight increase in breakup time. Thus, exploitation of citrate in potentially improving rheological properties of residual natural saliva or artificial preparations would not necessarily require citric acid concentrations sufficiently high to pose a risk to teeth.

To summarise, salivary rheology is clearly a multifactorial property. Mimicking the physical properties of saliva as a therapy for xerostomia or hyposalivation has proved challenging and is clearly not dependent on the presence of mucins alone. This work suggests that of equal if not greater importance than mucins themselves in facilitating salivary rheology are other salivary proteins (amylase and statherin) and endogenous metabolites such as citrate, both of which may interact with mucins and the ions in their local environment and modulate their physical state. Future approaches to understanding the rheology of saliva involving analysis of multiple salivary components may enable an improvement on current saliva substitutes. This could be, for example, by combining stimulants of residual salivary capacity (i.e. capsaicin) into artificial saliva formulations, combining artificial saliva with stimulated residual natural saliva. Developing mechanisms of preserving mucins in a state similar to their pre‐secretion configuration and allowing activation on demand may be possible. Finally, if defects in citrate transport were to be demonstrated in xerostomic individuals, developments in pharmaceuticals targeting SLC transporters may offer improvement.

## COMPETING INTERESTS

The authors declare no competing interests.

## AUTHOR CONTRIBUTIONS

All authors contributed to the study design, data analysis and interpretation. A.G. conducted the experiments. All authors have read and approved the final version of this manuscript and agree to be accountable for all aspects of the work in ensuring that questions related to the accuracy or integrity of any part of the work are appropriately investigated and resolved. All persons designated as authors qualify for authorship, and all those who qualify for authorship are listed.
